# Influence of Texture on Impact Toughness of Ferritic Fe-20Cr-5Al Oxide Dispersion Strengthened Steel

**DOI:** 10.3390/ma10070745

**Published:** 2017-07-03

**Authors:** Javier Sánchez-Gutiérrez, Jesus Chao, Javier Vivas, Francisco Galvez, Carlos Capdevila

**Affiliations:** 1 Materiales Estructurales Avanzados y Nanomateriales, Ciencia de Materiales, E.T.S. de Ingenieros de Caminos Canales y Puertos, Universidad Politecnica de Madrid, 28040 Madrid, Spain; javisangu27@gmail.com (J.S.-G.); fgalvez@mater.upm.es (F.G.); 2 Materalia Research Group, Centro Nacional de Investigaciones Metalurgicas, Consejo Superior Investigaciones Científicas (CENIM CSIC), 28006 Madrid, Spain; jchao@cenim.csic.es (J.C.); jvm@cenim.csic.es (J.V.)

**Keywords:** impact toughness behavior, delamination, mechanical alloying, oxide dispersion strengthened ferritic alloy

## Abstract

Fe-based oxide dispersion strengthened (ODS) steels are oriented to applications where high operating temperatures and good corrosion resistance is paramount. However, their use is compromised by their fracture toughness, which is lower than other competing ferritic-martenstic steels. In addition, the route required in manufacturing these alloys generates texture in the material, which induces a strong anisotropy in properties. The V-notched Charpy tests carried out on these alloys, to evaluate their impact toughness, reveal that delaminations do not follow the path that would be expected. There are many hypotheses about what triggers these delaminations, but the most accepted is that the joint action of particles in the grain boundaries, texture induced in the manufacturing process, and the actual microstructure of these alloys are responsible. In this paper we focused on the actual role of crystallographic texture on impact toughness in these materials. A finite elements simulation is carried out to solely analyze the role of texture and eliminate other factors, such as grain boundaries and the dispersed particles. The work allows us to conclude that crystallographic texture plays an important role in the distribution of stresses in the Charpy specimens. The observed delaminations might be explained on the basis that the crack in the grain, causing the delamination, is directly related to the shear stresses τ_12_ on both sides of the grain boundary, while the main crack propagation is a consequence of the normal stress to the crack.

## 1. Introduction

FeCrAl oxide dispersion-strengthened (ODS) alloys have wide-ranging applications, mostly in circumstances where high creep strength and oxidation resistance is a design criteria requirement at temperatures above 1000 °C. Typical applications include use in furnace construction as shields or carrier systems; in the glass industry as stirrers or plungers in molten glass; in the combustion of waste materials; as thermocouple protection tubes; in high temperature testing equipment; as burner tubes; and in a variety of applications in automotive diesel engines [1,2,3].

There are also potential applications of Fe-based ODS alloys in the nuclear power generation industry. Compared to austenitic alloys, ferritic-martensitic steels display higher thermal conductivity, a lower thermal expansion, and a lower tendency to He-embrittlement. The ferritic state makes them less susceptible to radiation-induced swelling. Creep properties of conventional ferritic or ferritic-martensitic (FM) steels are not sufficient to withstand the levels of mechanical loading reached in some core structures. For instance, at the end of life, for sodium cooled fast reactors (SFRs), the internal pressure in the cladding tubes could reach almost 100 MPa and the FM steels have to be reinforced. In this sense, Fe-based ODS alloys such as MA957 [4] were designed for use in a liquid sodium environment, at temperatures of the order of 700 °C. Both have a high void swelling resistance and a low carbon concentration, in order to avoid the formation of titanium carbides.

Nevertheless, the ferritic and/or FM ODS steels still present a drawback regarding the toughness behavior. Ukai et al. [5] demonstrated the importance of inclusion control in ODS steel fabrication: mechanical strength and toughness of an ODS steel can be largely improved by the fabrication process. Tanno et al. reported Charpy impact test results of premixed and fully prealloyed 11Cr-ODS steels [6]. It is clearly seen that impact properties are much improved in fully prealloyed steel, compared to those of premixed steel (with approximately four times the upper-shelf energy (USE), and with an apparently lower ductile-to-brittle transition temperature (DBTT)). This improvement is caused by a reduction of inclusions as apparently seen in fractographic examination. Besides the cleanliness of the steel production, other factors such as grain boundary segregation [1] and the texture of the material [7,8,9] will affect the toughness of the ODS. Bearing in mind that the conventional production route involves hot-extrusion, the resulting microstructure in ferritic ODS steels presents a crystallographic texture with an intense α-fibre [10,11]. This leads to strong anisotropy (in terms of mechanical properties), which also affects the toughness. All these results lead us to conclude that toughness in ferritic ODS steels depends on multiple factors, which should not be only the chemical compositions and fabrication process, but also the impurity level and crystallographic texture. In this paper, we try to solely disclose the role of crystallographic texture on Charpy impact toughness of ferritic ODS steel over other microstructural factors, such as effective grain size and oxide distribution. 

## 2. Materials and Techniques

Oxide dispersion strengthened (ODS) Fe20Cr6Al0.5Y_2_O_3_ alloy was commercially available as PM 2000™ and manufactured by Plansee GmbH, as reported elsewhere [12,13]. It is a ferritic alloy containing ~20 wt. % Cr and 5% Al for improving oxidation and corrosion resistance. The detailed chemical composition of the commercial purity PM 2000™ used in this study (as determined by X-ray Fluorescence (in a Bruker S8 Tiger Wave Length X-ray Fluorescence (WDXRF) spectrometer, Brucker, Karlsruhe, Germany)) is given in Table 1. After mechanical alloying, the alloyed powder is canned and hot-rolled to form a final 100 mm in diameter tube, with wall-thickness of 8 mm.

**Figure 1:**
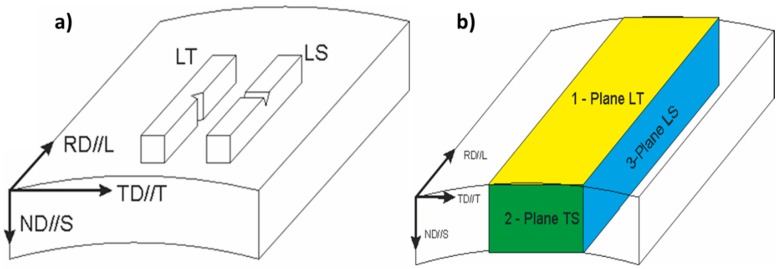
(**a**) Scheme indicating the position of the notch in respect to the rolling direction material; and (**b**) Scheme of sectioned samples for Electron Backscattering Diffraction (EBSD) measurements, respectively.

The orientation of the impact specimens are named following ASTM-399 standard by two letters: the first letter designates the direction normal to the crack plane, and the second letter designates the direction parallel to the striking direction (Figure 1a). L (longitudinal) direction is parallel to the rolling direction RD, T (transverse) is parallel to TD (transverse direction), and S (short transverse) direction is parallel to ND (normal direction). Charpy specimens of 55 × 6.2 × 6.2 mm^3^ with a V-notch of 1.25 mm in depth were mechanized in the LT and LS orientation. These reduced-section Charpy specimens were subjected to impact energy of 147 J and velocity at the contact instant of 5.4 m s^−1^, in a range of temperatures between −196 °C and 425 °C.

Microtexture analysis of the as-hot rolled PM 2000 tube specimens was performed by the Electron Backscattering Diffraction (EBSD) technique. EBSD patterns were collected at various locations on cross and flat sections, and carefully polished with colloidal silica (50 nm particle size) in the final stage. An ultrasonic cleaning process in ethanol at 30 °C is performed. The EBSD patterns were generated at an acceleration voltage of 20 kV and collected using a CRYSTAL detector of Oxford Instruments, mounted in a SEM JEOL JSM 6300 (JEOL, Tokyo, Japan). The index of the Kikuchi lines and the determination of the orientations were done with the software CHANNEL 5, developed by HKL Technology (Oxford, UK). The results were represented by means of inverse pole figure (IPF) maps, which give the orientation of a macroscopic direction with respect to a specific crystal direction. Samples were sectioned according to the scheme shown in Figure 1b. The rolling direction (RD) is indicated in the figure.

An elastoplastic Finite Element Modeling (FEM), performed by the multipurpose finite element program ABAQUS (provided by Universidad Politécnica de Madrid), was used to discern the effect of the residual stress on the Charpy impact toughness. The results were analyzed, comparing the aspect of the fracture surface of the Charpy samples with those of the shear (τ) iso-stress contours. For the sake of a good compromise between computational cost and reality, the following simulation consists of four fundamental elements: V-notched Charpy sample, striker, and two supports. All were designed as deformable 3D solids. All the pieces are designed according to the European standard EN-10 045-1. The V-notched sample dimension simulated was 55 × 10 × 10 mm^3^, while the notch angle was 45° and the dimension underneath the notch was 8 mm. The distance between supports considered was 40 mm. The width of the striker was 18 mm, with an angle of 30° and 2 mm tip radius.

## 3. FEM Simulation

The external reference system considered (Ex or Abaqus reference system) was designated as X, Y, and Z, where X|| [100], Y|| [010], and Z|| [001]. On the other hand, the internal reference system of the cubic crystal (Int) is x|| [100], y|| [110], and z|| [111]. The Young modulus (E), shear modulus (G), and Poisson’s modulus (ν) under the orthotropic assumption might be described by the following expressions [14]:

(1) $$\frac{1}{E}=S_{11}-2\left(S_{11}-S_{12}-\frac{1}{2}S_{44}\right)\left(l^{2}m^{2}+m^{2}n^{2}+n^{2}l^{2}\right)$$

(2) $$\frac{1}{G}=S_{44}+4\left(S_{11}-S_{12}-\frac{1}{2}S_{44}\right)\left(l^{2}m^{2}+m^{2}n^{2}+n^{2}l^{2}\right)$$

(3) $$\nu =-\frac{S_{12}+\left(S_{11}-S_{12}-\frac{1}{2}S_{44}\right)\left(l^{2}m^{2}+m^{2}n^{2}+n^{2}l^{2}\right)}{S_{11}-2\left(S_{11}-S_{12}-\frac{1}{2}S_{44}\right)\left(l^{2}m^{2}+m^{2}n^{2}+n^{2}l^{2}\right)}$$

The values of elastic constants for iron BCC considered are S_11_ = 131.2 GPa^−1^, S_12_ = −358.4 GPa^−1^, and S_44_ = 116.5 GPa^−1^ [15]. Besides, the (l,m,n) direction cosines between the Ex and Int reference systems are listed in Table 2. Therefore, the values of E, G, and ν for each of the crystallographic directions [100], [110], and [111] in iron BCC crystal are listed in Table 3.

Moreover, the specimen has elastic anisotropy (orthotropic material), but isotropic plasticity is assumed (Von Mises). However, the processing of this type of material is by hot-extrusion. Therefore, the flow of the material is not isotropic but occurring in extrusion direction (x-axis). The fact is that extrusion will force that larger amount of grain boundaries that are running parallel with the x-axis, and will deviate from the Von Mises stress distribution (which its isotropic character will miss (the anisotropy of the grain boundary movement)). For this reason, the stress analysis described below is focused on the τ_12_ shear stress component distribution instead. 

The simulation does not consider the fracture. However, during high-strain conditions, it was considered geometric nonlinearity, due to changes in geometry during analysis (i.e., large displacement analysis, in which the deformed geometry will be taken into the next calculation step).

Both the kicker and the supports are considered as linear elastic isotropic materials. For a simplified analysis, it was considered isotropic plasticity.

## 4. Results and Discussion

The anisotropy in the mechanical properties of ODS alloys, which is considered as structural material for different components in future nuclear power plants, compromises the optimum design of such components [16]. As it has been reported in literature [17,18,19], the aligned inclusions, elongated grains, and crystallographic texture are all factors that aggravate the orientation dependence of mechanical properties. In this sense, crystallographic texture can elucidate the role that cleavage planes (being parallel to the fracture plane of the Charpy specimens) have on increasing anisotropy [20], or bias the slip systems available for ductile fracture [21]. However, there is some discussion of the actual role of crystallography, because of the difficulty in separating individual causes of anisotropy. Some studies indicate a lack of correlation between the texture and orientation dependence of the Charpy properties [20,22]. This fact leads us to use the simulation approach to disclose if crystallographic texture plays a role in delamination, and it would be established that the anisotropy of the Charpy properties can be attributed, at a certain level, to the crystallographic texture.

### 4.1. Microstructure

**Figure 2:**
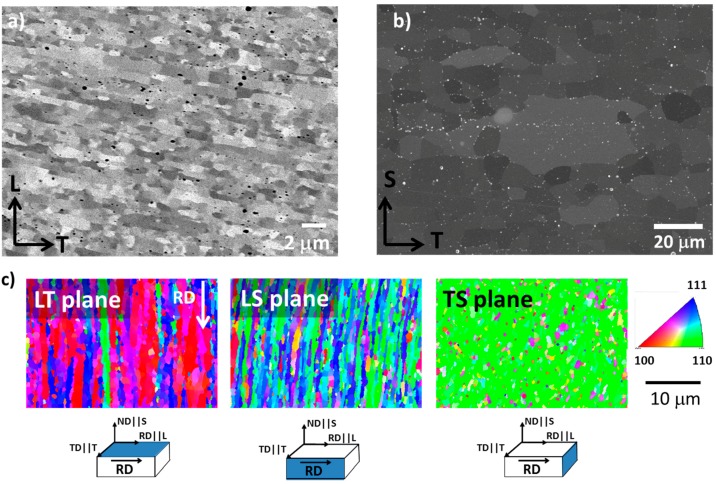
(**a**) Backscattered SEM of Longitudinal-Short transverse plane (LS) for the hot rolled condition; (**b**) secondary electron image of grain boundary particles (in white) in Transversal-Short transverse plane (TS) plane; and (**c**) (inverse pole figure) IPF maps taken in normal direction to means Longitudinal-Trasnverse plane (LT), LS, and TS planes, as shown in Figure 1.

The microstructures, after hot-rolling in the LT and TS planes, are shown in Figure 2a,b, respectively. The microstructure consists in elongated grains of 2 µm in length along RD, with almost equiaxed section of ~0.5 µm in diameter in the TS plane. A grain aspect ratio (GAR) of 4.4 was measured for the grains of the LT plane. As described elsewhere [23], the ODS particle reinforcement (i.e., complex Y-Al oxides, with sizes ranging from 3 to 40 nm) are preferentially located at the ferrite grain boundaries. Besides these oxide particles, numerous large inclusions with complex compositions were found throughout the sample, which preferentially form large stringers in the LS plane. The investigations showed those stringers are formed by large γ-Al_2_O_3_ inclusions, as well as complex γ-Al_2_O_3_/Y-Al-O impurities, which may additionally enclose smaller Ti(C,N) particles [3].

EBSD analyses were performed to determine the microtexture in the LT, TS, and LS planes. The IPF maps obtained are shown in Figure 2c, which indicate the <UVW> directions that are parallel to the Z-direction of the sample reference system. Texture analyses reveal that more than 80% of indexed grains present the <110> direction parallel to the RD, i.e., there is a major presence of (110) planes in the TS. No {100} cleavage planes were detected in the TS plane. This texture is typical of body-centered cubic materials, deformed by either extrusion or rolling. It is clear from Figure 2c that ND || <100>, i.e., the (100) plane, is parallel to the sheet tube surface. Likewise, this figure also indicates that the RD || <110> and the RD plane not only contain the <110> direction, but also the <111>. Therefore, it could be concluded that the as-received microstructure presents a strong texture with RD || <110> and ND || <100>.

### 4.2. Impact Toughness Results

**Figure 3:**
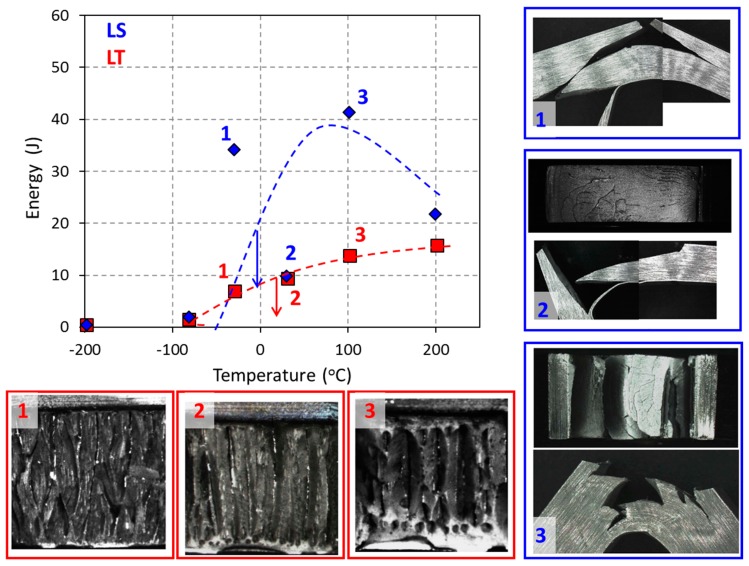
Charpy Impact energy of the PMT samples for LT and LS specimens. The arrows indicate Ductile-brittle transition temperature (DBTT) for LS and LT samples.

**Figure 4:**
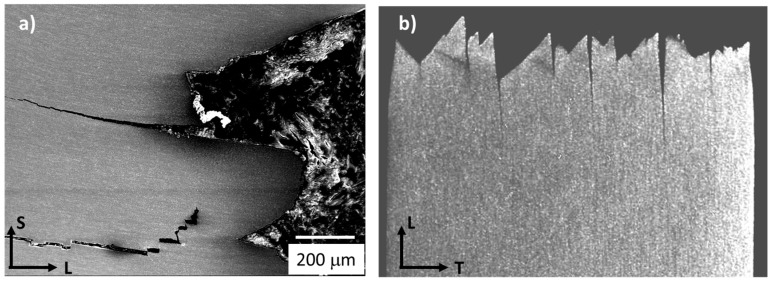
Details of delaminations in (**a**) LS and (**b**) LT specimens. Axes are indicated in the figure.

The impact fracture behaviour of both LT and LS specimens (of the PM 2000 alloy) has been reported elsewhere [24]. As a general overview, the impact energy results along with the corresponding fracture surfaces (in the TS plane) are presented in Figure 3 for the LT and LS notch configuration, indicated in Figure 1. These results point out the considerably higher absorbed energy in the high temperatures range, obtained by LS specimens as compared with LT specimens; however, the lower shelf energy does not vary much with the orientation of the notch. Figure 4 shows a detailed image of the delaminations produced in a LS sample (Figure 4a) and in a LT sample (Figure 4b). Bearing in mind the grain morphology shown in Figure 2, it might be reasonable to think that the crack progresses between grains, which could be caused by several factors. Alinger et al. [25] reported an anisotropic behavior in the fracture toughness of the hot-extruded MA957 ODS ferritic steels, and suggested that low toughness will be improved by the elimination of stringer-like alumina inclusions from impurities in the source powder. On the other hand, Kasada et al revealed the existence of elongated M_23_C_6_ in two types of hot-extruded ODS ferritic steels (Fe-19Cr-4Al-2W-0.3Ti-0.3Y_2_O_3_ and Fe-19Cr-0.3W-0.3Ti-0.3Y_2_O_3_), containing relatively high concentrations (0.05–0.09 wt. %) of impurity carbon [26]. All these results lead us to conclude that the morphology of brittle features in ODS ferritic steels depend on multiple factors, which should not be only the chemical compositions and fabrication process but also the impurity level and the situation of the raw powder. However, in the particular case studied here, the fractographic analysis carried out elsewhere [27] does not reveal the presence of oxides in the fractographic microstructure (that could come from the oxide stringers or particle alignment, observed Figure 2).

**Figure 5:**
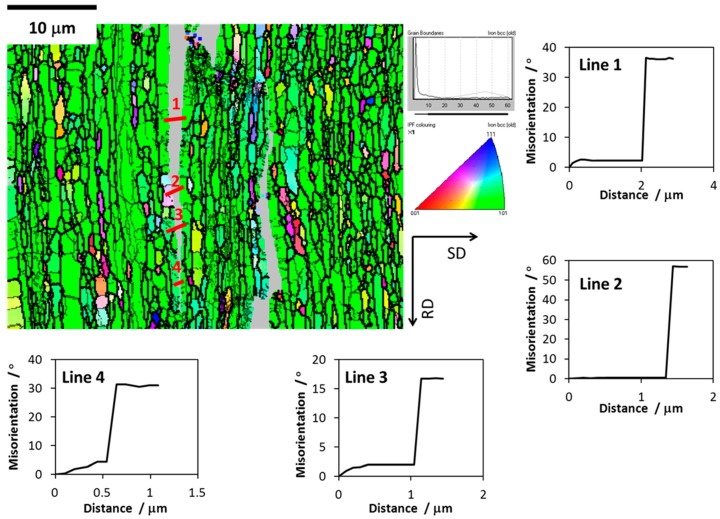
Misorientation analysis through lines across the crack in LT sample tested at room temperature. RD stands for rolling direction and SD for striking direction.

EBSD analysis was used to investigate the phenomena underlying the splitting in LT samples. Figure 5 illustrates the analyses performed in a plane parallel to the LS plane of LT samples (fractured at RT), which present a certain degree of delamination. The misorientations (Δθ) across the crack, between the numbered grains of every pair, were obtained. It indicates large misorientations across the crack. Thus, crack propagates along the grain boundaries during the delamination. The fracture surface indicates that the ductile component might be important; the cracks progress with a considerable amount of plastic deformation, and transverse to the RD, which is consistent with previous studies on laminated structures (reported by Kimura and co-workers) [28].

The presence of delaminations parallel to RD is something common in a variety of materials, such as thermomechanically treated steels [29,30], laminates [31], laminated composites [32], and FeCrAl ODS alloys [33]. It is a common characteristic in all the cases that inhomogeneities in some microstructural features induce the existence of splitting. If weak planes are parallel to the LT planes, the interactions of those weak planes with the stress field (generated by the localized plastic constrain at the notch and/or the crack tip) can cause splitting.

**Figure 6:**
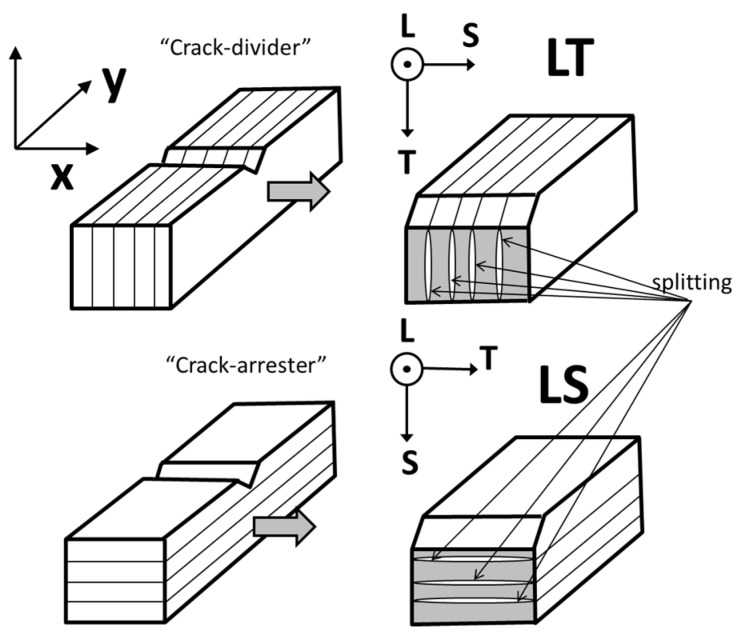
Schematic illustration showing laminate splitting geometries found in LT and LS samples tested, according with Cartesian x,y,z and L,T,S (Figure 1a) reference systems. (After Kimura and co-workers [28]).

There are two basic geometries termed “crack divider” and “crack arrester”, as schematically illustrated in Figure 6 [28]. In the case crack divider (LT specimens), the delaminations divide the specimen thickness in as many sub-specimens as delaminations. This causes a relaxation of the triaxiality stress state (at the notch tip, towards a biaxial stress state). 

On the contrary, in the case of the “crack arrester” (LS) specimens, the delamination causes a strong decrease in the stress intensity at the tip of the 90° bifurcated crack. The magnitude of this crack is about one half of that immediately before delamination. For further plastic strain, it is necessary to reinitiate the fracture across the unnotched ligament. This causes, as was observed, an apparent high toughening (Figure 3).

### 4.3. Role of Crystallographic Texture on Delamination

**Figure 7:**
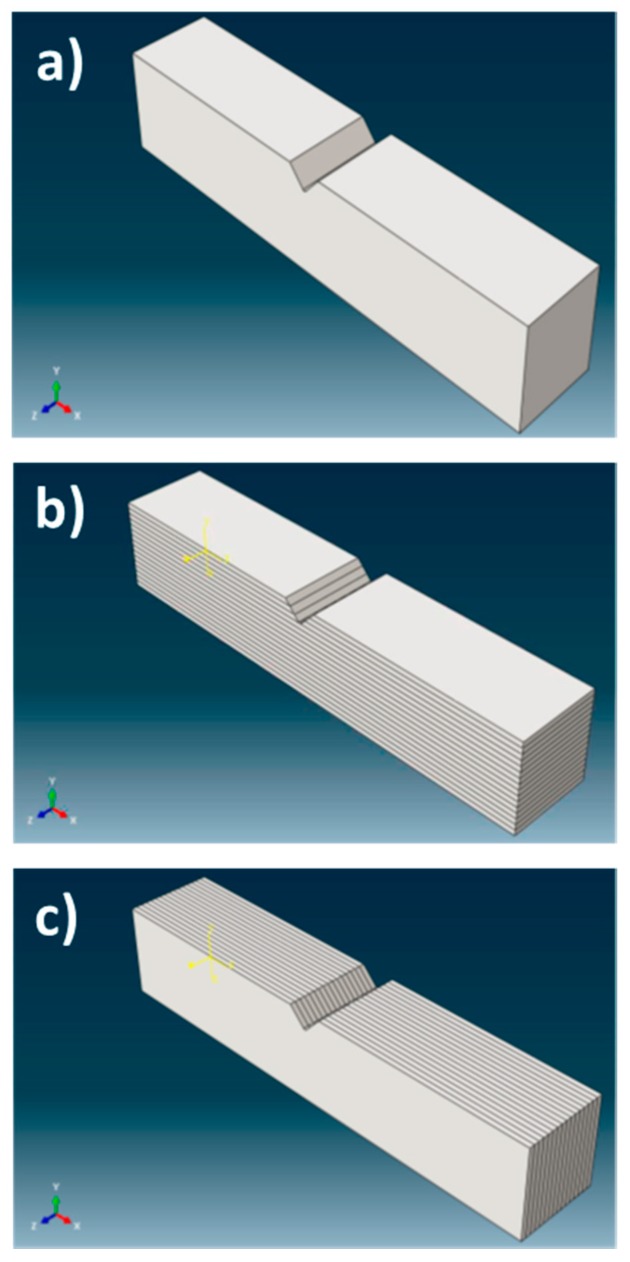
(**a**) Single crystal configuration; (**b**) arrester configuration; and (**c**) divider configuration.

The two configurations are related to the position of the notch relative to the Charpy sample, i.e., the “crack arrester” configuration (the notch is contained in the LT plane of the specimen), and the “crack divider” configuration (the notch is contained in the LS plane of the specimen), as shown in Figure 2. The effect of texture is introduced in the simulation, by means of partitions of the Charpy sample. Each partition (illustrated in Figure 7b,c, in contrast to the single crystal case shown in Figure 7a) simulates an elongated grain with a particular crystal orientation. Each partition presents a small twist (maximum ±12°), regarding its immediate neighbor to simulate the microtexture. This twist is introduced through the external reference system (Ex system). The rotations considered in each partition are listed in Table 4. These partitions are virtual, that is, the interface between both planes is assumed perfect, so the piece is a continuum. No grain “boundary effect”, understood as a transition between neighboring grains, is considered in the simulation.

**Figure 8:**
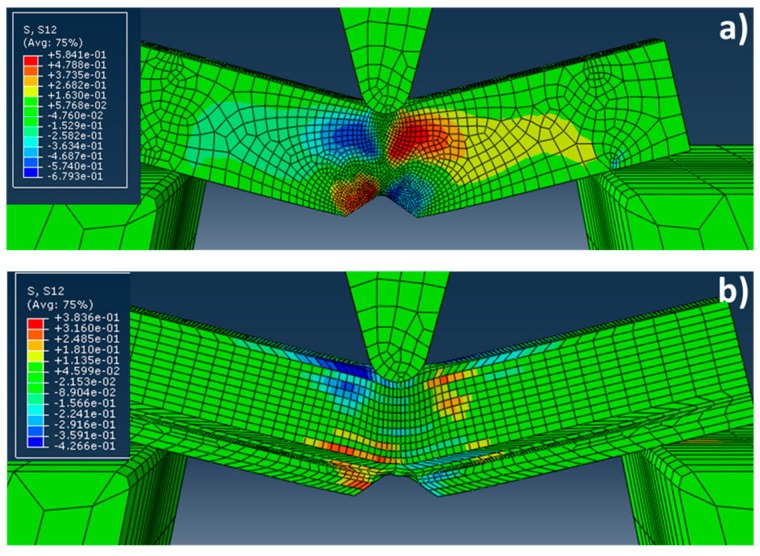
Lateral view (XY-plane) of the distribution of τ_12_ shear stresses during the Charpy test for: (**a**) the single crystal case; and (**b**) the arrester configuration.

Figure 8 shows the results obtained in the distribution of τ_12_ shear stresses for the single crystal case. It is observed in the Figure 8a that the τ_12_ shear stresses are homogeneously distributed in the XY-plane. As might be expected, the negative (in blue) and positive (in red) τ_12_ shear stresses are located symmetrically at different sides of the notch. By contrast, for the “crack arrester” configuration, it is observed how the τ_12_ shear stresses are located heterogeneously between the different elements that form the Charpy sample. 

**Figure 9:**
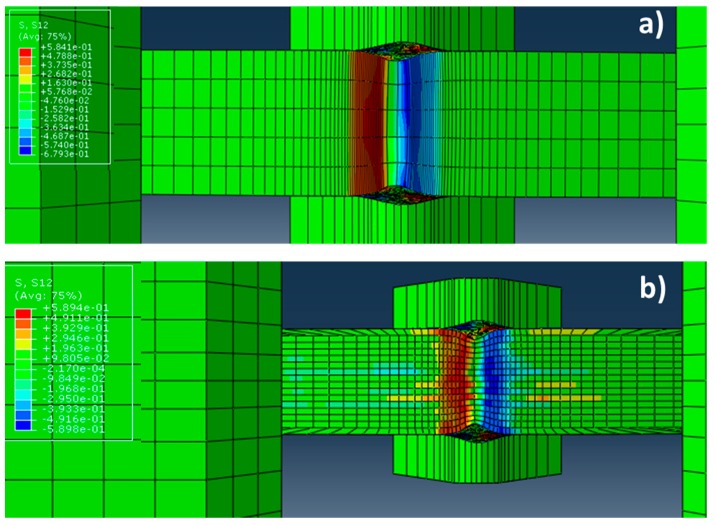
Distribution of τ_12_ shear stresses in a plane corresponding to the view from below (XZ-plane), during the Charpy test for: (**a**) the single crystal case; and (**b**) the divider configuration.

On the other hand, Figure 9 illustrates the distribution of the τ_12_ shear stresses in a plane, corresponding to the view from below during the Charpy test (XZ-plane). As in the above case, the negative (in blue) and positive (in red) τ_12_ shear stresses are located symmetrically at different sides of the notch for the single crystal case, but some heterogeneous distributions are observed for the divider configuration. However, it is less pronounced that in the case of the “crack arrester” configuration.

By comparing those results and the experimental ones shown in Figure 3, it might be concluded that residual shear stresses are accumulated at the grain boundaries, due to the texture of the material. Since the different elements considered in the modelling are misoriented only a few degrees (between 3° and 14°), it could be considered that the materials with both arrester and divider configurations present a strong texture, with elongated “grains” with small angle boundaries. The delaminations shown in Figure 3 are consistent with the interpretation of crack nucleated at the grain boundaries, because of shear stress concentration (due to texture misorientation).

## 5. Conclusions

The main conclusion of this work is the verification of the role of the crystallographic texture on the distribution of stresses in the Charpy specimens. To explain the observed delaminations, we have assumed that the nucleation of the crack at the grain boundary (causing the delamination) is directly related to the shear stresses τ_12_ on both sides of the grain, while the main crack propagation is a consequence of the normal stress to the crack.

Both the crack-arrester and crack-divider configurations have a shear stress distribution τ_12_ that allows the assertion that there is a shear deformation between neighboring partitions in the model (which would correspond to shear deformations between neighboring grains). These stresses could explain why intergranular cracks, inducing delaminations, are nucleated in the Charpy tests.

## Figures and Tables

**Table 1:** Chemical composition of PM 2000, as determined by X-Ray Fluorescence.

Composition	Cr	Al	Ti	C	O	N	Y	Fe
wt. %	18.60	5.20	0.54	0.04	0.09	0.006	0.391	balance
at. %	18.50	10.10	0.58	0.17	0.28	0.022	0.228	balance

**Table 2:** Direction cosines.

Reference System	[100]	[110]	[111]
X[100]	L = 1	$l=1/\sqrt{2}$	$l=1/\sqrt{3}$
Y[010]	M = 0	$m=1/\sqrt{2}$	$m=1/\sqrt{3}$
Z[001]	N = 0	N = 0	$n=1/\sqrt{3}$

**Table 3:** Direction-dependent values of Young Modulus (E_ii_), Shear Modulus (G_ij_), and Poisson coefficient (ν_ij_).

E_ii_ (GPa)	G_ij_ (GPa)	ν_ij_
$E_{11}=131$	$G_{12}=116$	$\nu_{12}=0.366$
$E_{22}=219$	$G_{13}=68$	$\nu_{13}=0.276$
$E_{33}=283$	$G_{23}=60$	$\nu_{23}=0.212$

**Table 4:** Twist implemented at each partition for the “crack arrester” and “crack divider” configurations.

Partition #	Twist (°)
1	−11
2	1
3	−7
4	−5
5	2
6	4
7	11
8	0
9	−10
10	3
11	−9
12	3
13	−5
14	1
15	−3
16	2

## References

[1] Miodownik M.A., Martin J.W., Little E.A. (1993). Grain boundary segregation in an oxide-dispersion-strengthened ferritic steel. J. Mater. Sci. Lett..

[2] Hack G.A.J. (1984). Developments in the production of oxide dispersion strengthened superalloys. Powder Metall..

[3] Capdevila C., Miller M.K., Toda I., Chao J. (2010). Influence of the α-α’ phase separation on the tensile properties of fe-base ODS PM 2000 alloy. Mater. Sci. Eng. A.

[4] Huet J.J. (1967). Possible fast-reactor canning material strengthened and stabilized by dispersion. Powder Metall..

[5] Ukai S., Ohtsuka S., Kaito T., de Carlan Y., Ribis J., Malaplate J., Yvon P. (2016). Oxide dispersion-strengthened/ferrite-martensite steels as core materials for generation IV nuclear reactors. Structural Materials for Generation IV Nuclear Reactors.

[6] Tanno T., Ohtsuka S., Yano Y., Kaito T., Tanaka K. (2014). Effects of manufacturing process on impact properties and microstructures of ODS steels. J. Nucl. Mater..

[7] Chao J., Capdevila C. (2014). Anisotropy in mechanical properties and fracture behavior of an oxide dispersion fe20cr5al alloy. Metall. Mater. Trans. A Phys. Metall. Mater. Sci..

[8] Chao J., Capdevila C., Serrano M., Garcia-Junceda A., Jimenez J.A., Miller M.K. (2014). Effect of α-α’ phase separation on notch impact behavior of oxide dispersion strengthened (ODS) fe20cr5al alloy. Mater. Des..

[9] Chao J., Rementeria R., Aranda M., Capdevila C., Gonzalez-Carrasco J.L. (2016). Comparison of ductile-to-brittle transition behavior in two similar ferritic oxide dispersion strengthened alloys. Materials.

[10] Pimentel G., Aranda M.M., Chao J., González-Carrasco J.L., Capdevila C. (2015). Development of simultaneous corrosion barrier and optimized microstructure in fecral heat-resistant alloy for energy applications. Part II: The optimized creep-resistant microstructure. JOM.

[11] Pimentel G., Aranda M.M., Chao J., González-Carrasco J.L., Capdevila C. (2015). Development of simultaneous corrosion barrier and optimized microstructure in fecral heat-resistant alloy for energy applications. Part 1: The protective scale. JOM.

[12] Capdevila C., Miller U., Jelenak H., Bhadeshia H. (2001). Strain heterogeneity and the production of coarse grains in mechanically alloyed iron-based PM 2000 alloy. Mater. Sci. Eng. A-Struct. Mater. Prop. Microstruct. Process..

[13] Capdevila C., Miller M., Russell K. (2008). Aluminum partitioning during phase separation in Fe–20%Cr–6%Al ODS alloy. J. Mater. Sci..

[14] Date E.H.F., Andrews K.W. (1969). Anisotropic and composition effects in the elastic properties of polycrystalline metals. J. Phys. D Appl. Phys..

[15] Lord A.E., Beshers D.N. (1965). Elastic stiffness coefficients of iron from 77° to 673° k. J. Appl. Phys..

[16] De Bremaecker A. (2012). Past research and fabrication conducted at SCK·CEN on ferritic ODS alloys used as cladding for fbr’s fuel pins. J. Nucl. Mater..

[17] Mintz B., Morrison W.B., Morris P.P., Davies G.J., Davies G.J. (1976). The influence of texture on the tensile and impact properties of controlled steels. Texture and Properties of Materials.

[18] Mintz B., Morrison W.B., Welch P.I., Davies G.J., Gottstein G., Lucke K. (1978). The relative contributions of texture and grain shape to the properties of warm-rolled Fe–Mn alloys. Texture and Properties of Materials.

[19] Petrov R., García O.L., Mouriño N.S., Kestens L., Bae J.H., Kang K.B. (2007). Microstructure-texture related toughness anisotropy of api-x80 pipeline steel characterized by means of 3d-ebsd technique. Mater. Sci. Forum.

[20] Petrov R., Garcia O.L., Mulders J.J.L., Reis A.C.C., Bae J.H., Kestens L., Houbaert Y. (2007). Three dimensional microstructure-microtexture characterization of pipeline steel. Mater. Sci. Forum.

[21] Baczynski G.J., Jonas J.J., Collins L.E. (1999). The influence of rolling practice on notch toughness and texture development in high-strength linepipe. Metall. Mater. Trans. A Phys. Metall. Mater. Sci..

[22] García O.L., Petrov R., Bae J.H., Kestens L., Kang K.B. (2007). Microstructure—Texture related toughness anisotropy of api-x80 pipeline steel. Adv. Mater. Res..

[23] Pimentel G., Toda-Caraballo I., Chao J., Capdevila C. (2012). Role of strain heterogeneity on recrystallisation of oxide dispersion strengthened fe-cr-al alloys for high-temperature applications. J. Mater. Sci..

[24] Chao J., Capdevila C., Serrano M., Garcia-Junceda A., Jimenez J.A., Pimentel G., Urones-Garrote E. (2013). Notch impact behavior of oxide-dispersion-strengthened (ODS) Fe20Cr5al alloy. Metall. Mater. Trans. A Phys. Metall. Mater. Sci..

[25] Alinger M.J., Odette G.R., Lucas G.E. (2002). Tensile and fracture toughness properties of ma957: Implications to the development of nanocomposited ferritic alloys. J. Nucl. Mater..

[26] Kasada R., Toda N., Yutani K., Cho H.S., Kishimoto H., Kimura A. (2007). Pre- and post-deformation microstructures of oxide dispersion strengthened ferritic steels. J. Nucl. Mater..

[27] Chao J., Capdevila-Montes C., González-Carrasco J.L. (2009). On the delamination of fecral ODS alloys. Mater. Sci. Eng. A.

[28] Kimura Y., Inoue T., Fuxing Y.I.N., Tsuzaki K. (2010). Delamination toughening of ultrafine grain structure steels processed through tempforming at elevated temperatures. ISIJ Int..

[29] Tsuji N., Okuno S., Koizumi Y., Minamino Y. (2004). Toughness of ultrafine grained ferritic steels fabricated by arb and annealing process. Mater. Trans..

[30] Song R., Ponge D., Raabe D. (2005). Mechanical properties of an ultrafine grained C-Mn steel processed by warm deformation and annealing. Acta Mater..

[31] Douthwaite R.M., Evans J.T., Petch N.J., Wraith A.E. (1974). Pneumatic bursting of multiple-layer pressure shells. Metals Technol..

[32] Kum D.W., Oyama T., Wadsworth J., Sherby O.D. (1983). The impact properties of laminated composites containing ultrahigh carbon (UHC) steels. J. Mech. Phys. Solids.

[33] Tomita Y. (2000). Development of fracture toughness of ultrahigh strength, medium carbon, low alloy steels for aerospace applications. Int. Mater. Rev..

